# Supplementary material for: In-depth secretome analysis of Puccinia striiformis f. sp. tritici in infected wheat uncovers effector functions
Source: Biosci Rep. 2020 Dec 4;40(12):BSR20201188. doi: 10.1042/BSR20201188 (PMC7724613; doi:10.1042/BSR20201188)
Supplement: Supplementary Figures S1-S3 [file BSR-2020-1188_supp.pdf]

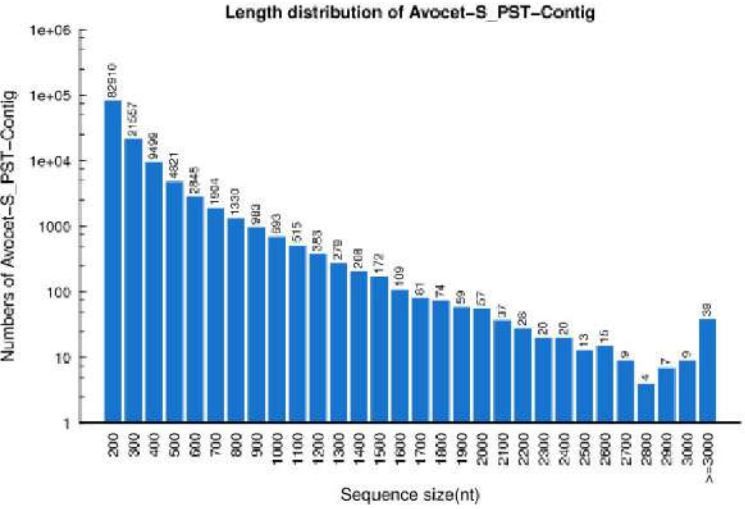
Length distribution of Avocet-S_PST-Contig
Numbers of Avocet-S_PST-Contig
Sequence size(nt)

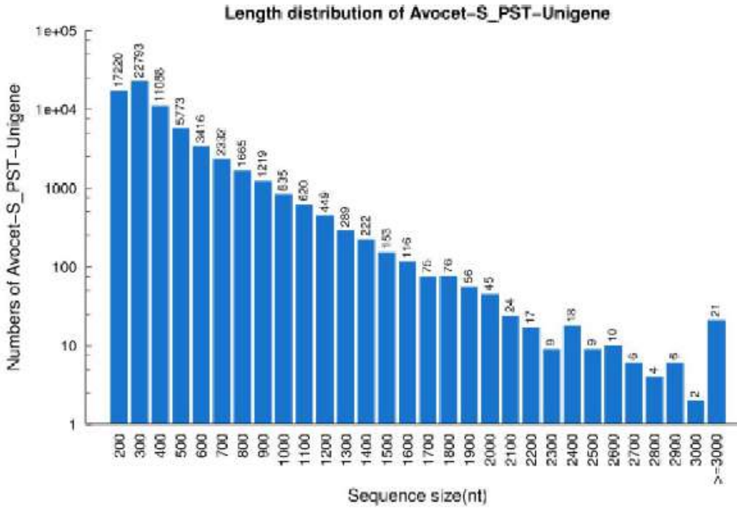
Length distribution of Avocet-S_PST-Unigene
Numbers of Avocet-S_PST-Unigene
Sequence size(nt)

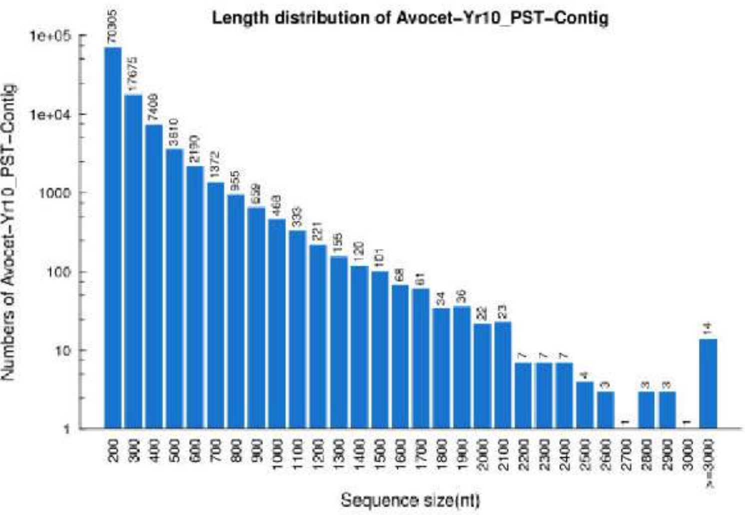
Length distribution of Avocet-Yr10_PST-Contig
Numbers of Avocet-Yr10_PST-Contig
Sequence size(nt)

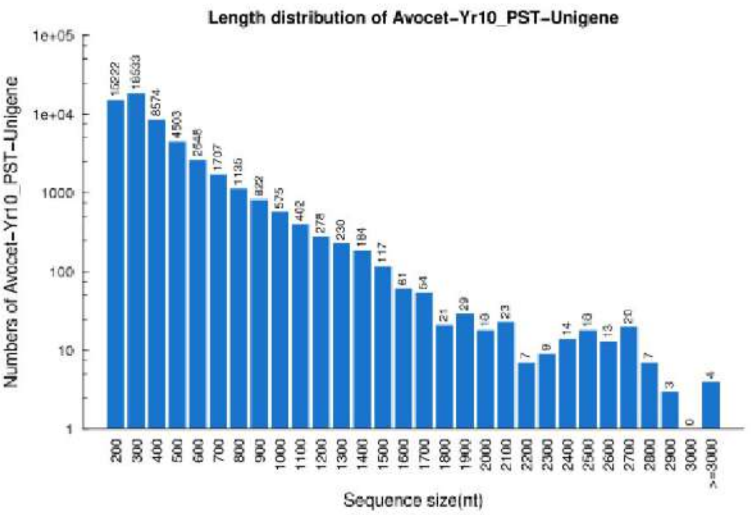
Length distribution of Avocet-Yr10_PST-Unigene
Numbers of Avocet-Yr10_PST-Unigene
Sequence size(nt)

**Figure S1. Length distribution of contigs and unigenes after the assembly process.** Graphical representation of the length distribution of A) contigs of AvocetS_PST, B) unigenes of AvocetS_PST, C) contigs of AvocetYR10_PST and D) unigenes of AvocetS_PST.

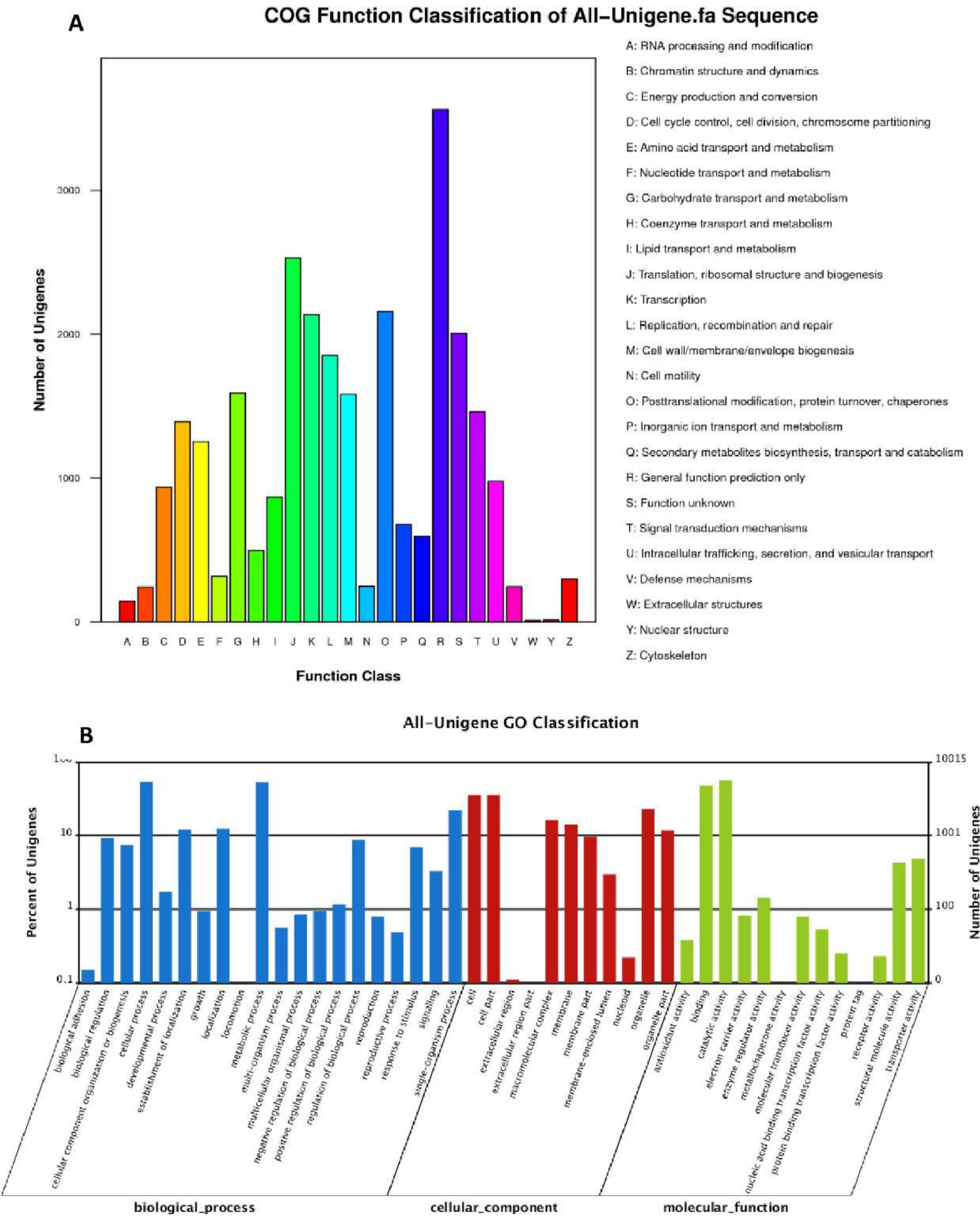
A
COG Function Classification of All-Unigene.fa Sequence
Number of Unigenes
3000
2000
1000
0
A B C D E F G H I J K L M N O P Q R S T U V W Y Z
Function Class
A: RNA processing and modification
B: Chromatin structure and dynamics
C: Energy production and conversion
D: Cell cycle control, cell division, chromosome partitioning
E: Amino acid transport and metabolism
F: Nucleotide transport and metabolism
G: Carbohydrate transport and metabolism
H: Coenzyme transport and metabolism
I: Lipid transport and metabolism
J: Translation, ribosomal structure and biogenesis
K: Transcription
L: Replication, recombination and repair
M: Cell wall/membrane/envelope biogenesis
N: Cell motility
O: Posttranslational modification, protein turnover, chaperones
P: Inorganic ion transport and metabolism
Q: Secondary metabolites biosynthesis, transport and catabolism
R: General function prediction only
S: Function unknown
T: Signal transduction mechanisms
U: Intracellular trafficking, secretion, and vesicular transport
V: Defense mechanisms
W: Extracellular structures
Y: Nuclear structure
Z: Cytoskeleton
B
All-Unigene GO Classification
Percent of Unigenes
10
1
0.1
Number of Unigenes
10015
1001
100
0
biological adhesion
biological regulation
cellular component organization or biogenesis
cellular process
developmental process
establishment of localization
growth
localization
locomotion
metabolic process
multi-organism process
multicellular organismal process
negative regulation of biological process
positive regulation of biological process
regulation of biological process
reproduction
reproductive process
response to stimulus
signaling
single-organism process
cell
cell part
extracellular region
extracellular region part
macromolecular complex
membrane
membrane part
membrane-enclosed lumen
nucleoid
organelle
organelle part
antioxidant activity
binding
catalytic activity
electron carrier activity
enzyme regulator activity
metallochaperone activity
molecular transducer activity
nucleic acid binding transcription factor activity
protein binding transcription factor activity
protein tag
receptor activity
structural molecule activity
transporter activity
biological_process
cellular_component
molecular_function

**Figure S2. Function annotation of the unigene data.** The function of each of the unigeneswas determined using the A) COG or B) GO databases.

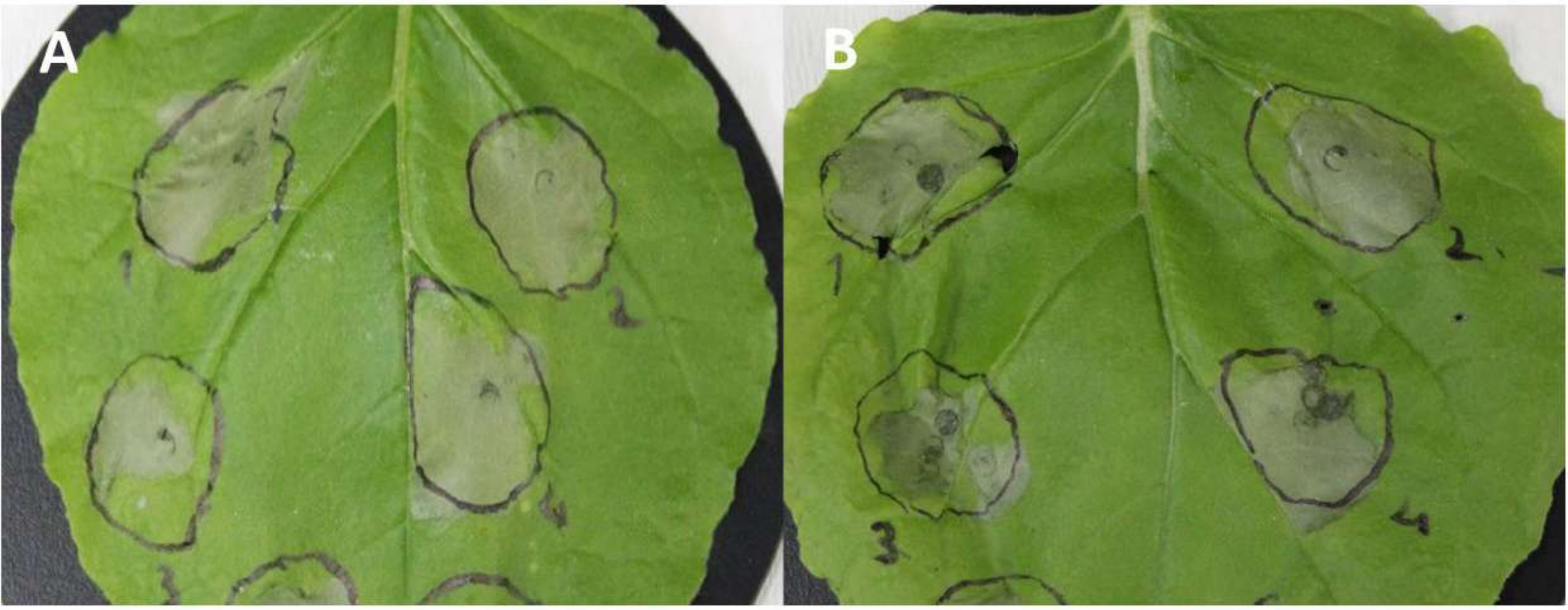
A
B

**Figure S3.** Photographs of the moderate and weak suppression phenotype of cell death mediated by Inf1 expression. (A) Moderate and (B) Weak suppression. Constructs were expressed with Agrobacterium tumefaciens; (1) GFP, (2) SP-GFP, (3) Unigene17495 without SP (Pstg10917ΔSP-GFP) and (4) Unigene17495 (Pstg10917-GFP). Cell death elicitor (Inf1) was infiltrated into the same region after 24 hours. Photos were taken at 4 days after Inf1 challenge.
