# Supplementary material for: In-depth secretome analysis of Puccinia striiformis f. sp. tritici in infected wheat uncovers effector functions
Source: Biosci Rep. 2020 Dec 4;40(12):BSR20201188. doi: 10.1042/BSR20201188 (PMC7724613; doi:10.1042/BSR20201188)
Supplement: Supplementary Table S1-S5 [file BSR-2020-1188_supp1.zip › BSR-2020-1188_suppST4.pdf]

Table S4. Matching PstDESSPs compared against YR candidates identified in Xia *et al*., 2017.

*Three of PstDESSPs overlapped with three previously neglected YR candidates in Xia et al., 2017.

Gene ID	Pucciniales homologs	Avr candidates
CL3094.Contig2_All	PSTG_09464T0*	YR6
CL6786.Contig1_All	PSTG_02003T0	YR6
Unigene30809_All	PSTG_03083T0	YRTR1
Unigene31162_All	PSTG_14206T0	YR9
Unigene31932_All	PSTG_08524T0	YR9
Unigene37241_All	PSTG_11923T0*	YR17
Unigene37856_All	PSTG_16854T0*	YR9
Unigene38264_All	PSTG_00708T0	YR6
Unigene7586_All	PSTG_14207T0	YR9
