# Supplementary material for: In-depth secretome analysis of Puccinia striiformis f. sp. tritici in infected wheat uncovers effector functions
Source: Biosci Rep. 2020 Dec 4;40(12):BSR20201188. doi: 10.1042/BSR20201188 (PMC7724613; doi:10.1042/BSR20201188)
Supplement: Supplementary Table S1-S5 [file BSR-2020-1188_supp1.zip › BSR-2020-1188_suppST5.pdf]

Table S5. Primers used in this study for the cloning.

Primer Names	Sequences (5'-3' direction)	Length (bp)
CACC-SP-917F	CACCATGTTGTTCTACGTTTACCTCA	26
CACC-917F	CACCATGCAGACTTTACCTTCCG	23
917Rev-STP	CTAGCATGTTTCCCAGCCTCC	21
917Rev	GCATGTTTCCCAGCCTCCG	19
